# Assessment of psychological impact and relationships interpersonal in women with malignant breast neoplasia

**DOI:** 10.1192/j.eurpsy.2024.1348

**Published:** 2024-08-27

**Authors:** M. C. V. R. De Oliveira, C. B. Soares de Oliveira, F. P. Vieira Silva, M. P. R. Camargo, N. Gonçalves de Sá

**Affiliations:** MEDICINE SCHOOL, UNOESTE, PRESIDENTE PRUDENTE, Brazil

## Abstract

**Introduction:**

Cancer causes an impact in the face of its news, whether due to feelings of anguish, stress and suffering due to the presence of the disease, which can be shared between patients, family members and loved ones. The news regarding the diagnosis generates, in addition to the psychological impact, financial difficulties, as the patient himself can often be responsible for a large part of the family income. Other complications are the difficulties in understanding the disease by the family members and/or the patient, denial of the disease in order to spare the patient from suffering and other loved ones, family conflicts related to the need to adapt to the new routine of daily life that the family should carry out aiming at the well-being of the patient and his treatment.

**Objectives:**

To evaluate the psychological impact and interpersonal relationships in patients with breast cancer treated in the city of Presidente PrudenteSP by a support association.

**Methods:**

This is an observational, quantitative, analytical and cross-sectional study, in which 200 patients with malignant breast cancer will be invited.

**Results:**

The sociodemographic results found were: 62.5% white women, 65.6% aged between 45-65 years, 56.3% married, 46.9% have completed higher education, 56.3% had no family history of cancer, predominance of stages II, III and IV when discovered, 93.5% did not drink, 84.4% did not smoke. On the anxiety scale, 53.1% and 43.8% report getting tired easily and feeling like crying, respectively. On the social adequacy scale, 72.5% continued working only with some limitation during treatment, despite this, 41.4% had minor financial difficulties, 34.5% had difficulties expressing feelings with family members, 40.7% had a relationship well with family members with small arguments and finally 34.8% felt affection for the partner all the time, despite this 36.4% did not have sexual intercourse with them in the last month.

**Conclusions:**

It was concluded, therefore, that when a family member gets sick, they all feel impacted, and each family will deal with the experience in a particular way, therefore, it is worth highlighting the encouragement of family participation in therapy sessions.

**Disclosure of Interest:**

None Declared

